# Pentobarbital versus thiopental in the treatment of refractory intracranial hypertension in patients with traumatic brain injury: a randomized controlled trial

**DOI:** 10.1186/cc6999

**Published:** 2008-08-29

**Authors:** Jon Pérez-Bárcena, Juan A Llompart-Pou, Javier Homar, Josep M Abadal, Joan M Raurich, Guillem Frontera, Marta Brell, Javier Ibáñez, Jordi Ibáñez

**Affiliations:** 1 Intensive Care Medicine Department, Son Dureta University Hospital, Andrea Doria 55, Palma de Mallorca, 07014, Spain; 2 Surgery Department, Universitat Autònoma de Barcelona (UAB), Bellaterra, 08193, Spain; 3 Investigation Unit, Son Dureta University Hospital, Andrea Doria 55, Palma de Mallorca, 07014, Spain; 4 Neurosurgery Department, Son Dureta University Hospital, Andrea Doria 55, Palma de Mallorca, 07014, Spain

## Abstract

**Introduction:**

Experimental research has demonstrated that the level of neuroprotection conferred by the various barbiturates is not equal. Until now no controlled studies have been conducted to compare their effectiveness, even though the Brain Trauma Foundation Guidelines recommend that such studies be undertaken. The objectives of the present study were to assess the effectiveness of pentobarbital and thiopental in terms of controlling refractory intracranial hypertension in patients with severe traumatic brain injury, and to evaluate the adverse effects of treatment.

**Methods:**

This was a prospective, randomized, cohort study comparing two treatments: pentobarbital and thiopental. Patients who had suffered a severe traumatic brain injury (Glasgow Coma Scale score after resuscitation ≤ 8 points or neurological deterioration during the first week after trauma) and with refractory intracranial hypertension (intracranial pressure > 20 mmHg) first-tier measures, in accordance with the Brain Trauma Foundation Guidelines.

**Results:**

A total of 44 patients (22 in each group) were included over a 5-year period. There were no statistically significant differences in ' baseline characteristics, except for admission computed cranial tomography characteristics, using the Traumatic Coma Data Bank classification. Uncontrollable intracranial pressure occurred in 11 patients (50%) in the thiopental treatment group and in 18 patients (82%) in the pentobarbital group (*P *= 0.03). Under logistic regression analysis – undertaken in an effort to adjust for the cranial tomography characteristics, which were unfavourable for pentobarbital – thiopental was more effective than pentobarbital in terms of controlling intracranial pressure (odds ratio = 5.1, 95% confidence interval 1.2 to 21.9; *P *= 0.027). There were no significant differences between the two groups with respect to the incidence of arterial hypotension or infection.

**Conclusions:**

Thiopental appeared to be more effective than pentobarbital in controlling intracranial hypertension refractory to first-tier measures. These findings should be interpreted with caution because of the imbalance in cranial tomography characteristics and the different dosages employed in the two arms of the study. The incidence of adverse effects was similar in both groups.

**Trial Registration:**

(Trial registration: US Clinical Trials registry NCT00622570.)

## Introduction

High dosages of barbiturates are used in patients with severe traumatic brain injury (TBI) who present with refractory intracranial hypertension (ICH) after medical and surgical treatment. This practice is recommended in the Brain Trauma Foundation (BTF) Guidelines, because this is the only second-level measure for which there is class II evidence that it can reduce intracranial pressure (ICP) [1]. Nevertheless, its effect on outcome is unproven [2], mainly because of severe medical complications.

Within the family of barbiturates the oxibarbiturates and thiobarbiturates stand out, their primary representatives being pentobarbital and thiopental. Until now no controlled studies have been reported that compare the effectiveness of pentobarbital and thiopental in controlling ICH. At the experimental level, research has demonstrated that mechanisms of action and levels of neuroprotection differ between these agents [3-6]. For this reason, research is needed to compare the effectiveness of these two drugs in terms of controlling refractory ICH in patients with severe TBI.

Based on various studies conducted in laboratory animals [3-6], suggesting that the neuroprotective capacity of thiopental is superior, our working hypothesis was that thiopental is more effective than pentobarbital in controlling ICP in patients with severe TBI, with a similar incidence of adverse side effects. In support of our work in the present study, the BTF Guidelines recommend that studies be undertaken to compare the effectiveness of the different barbiturates that are currently used in TBI patients [1].

## Materials and methods

We conducted a prospective, randomized cohort study comparing two treatments: pentobarbital and thiopental. Our primary objective was to compare the effectiveness of these agents in controlling refractory ICH in patients with severe TBI. Secondary objectives were to compare the incidence of secondary effects, especially arterial hypotension, which was defined as the presence of mean arterial pressure (MAP) under 80 mmHg at any point during barbiturate therapy.

This study was conducted at Son Dureta University Hospital (Palma de Mallorca, Spain) and was approved by the Ethics Committee of the Balearic Islands on 31 March 2002. It is registered with the US Clinical Trials Registry, with the number NCT00622570.

In all cases, the patient's closest relative, legal representative, or guardian gave written informed consent for their inclusion in the study.

### Inclusion criteria

Patients admitted to our intensive care unit (ICU) between May 2002 and July 2007 with a severe TBI (Glasgow Coma Scale [GCS] score after nonsurgical resuscitation ≤ 8) and presenting with refractory ICH (ICP > 20 mmHg), and who underwent first-level measures in accordance with the BTF Guidelines [7], were included. Refractory ICH was defined as follows: ICP 21 to 29 mmHg for 30 minutes or more, ICP of 30 to 39 mmHg for 15 minutes or more, or ICP greater than 40 mmHg for more than 1 minute, in the absence of external interventions. Included patients were required to be haemodynamically stable at the point of inclusion in the study; haemodynamic stability was defined as systolic blood pressure of 100 mmHg or greater.

### Exclusion criteria

We did not include in the study patients who were younger than 15 or older than 76 years; patients with a GCS score of 3 upon admission and neurological signs of brain death (bilateral arreactive midryasis and loss of brainstem reflexes); and patients who were pregnant, had barbiturate allergy or intolerance, or had a history of severe cardiac ventricular dysfunction with an ejection fraction under 35%.

### General therapeutic protocol

All patients with severe TBI underwent cranial computed tomography (CT) upon admission and were categorized in accordance with the classification proposed by the Traumatic Coma Data Bank [8]. We also recorded findings of CTs conducted before inclusion of the patients in the study. The CT findings on inclusion were regarded to be the worst of the hospital stay; the prognostic value of such CT findings have been described by other authors [9]. CTs were independently reviewed and categorized by two neurosurgeons (JI and MB) who were unaware of the treatment group to which the patients had been assigned. In cases of disagreement between these investigators, a third investigator reviewed the CT images.

All patients' ICP was monitored using an intraparenchymal Camino catheter (Integra Neurosciences, Plainsboro, NJ, USA). The ICP catheter was placed in the frontal region of the hemisphere with more radiological lesions on the CT. The systemic monitoring of these patients included invasive blood pressure, pulse oximetry, and a pulmonary artery thermodilution catheter. The ICP, MAP and cerebral perfusion pressure data were gathered on an hourly basis (one value every full hour) throughout the study using the Care Vue^® ^clinical monitoring system (Phillips, Eindhoven, The Netherlands).

The general treatment objectives in patients with severe TBI were to maintain MAP above 80 mmHg, ICP below 20 mmHg, and cerebral perfusion pressure above 60 mmHg. To achieve these objectives, we used liquids and/or vasoactive support with norepinephrine (noradrenaline).

In patients with ICP greater than 20 mmHg, initial treatment included elevation of the head of the bed, keeping the neck straight, appropriate sedation, muscular paralysis, ventricular drainage (if the patient had visible ventricles on the CT), 20% mannitol (0.25 to 0.75 mg/kg), 7.5% hypertonic saline (2 ml/kg) and moderate hyperventilation (partial carbon dioxide tension of 30 to 35 mmHg). Neurosurgical interventions were undertaken when necessary to evacuate surgical lesions. This approach can be considered conventional treatment and is included in the BTF Guidelines as first-tier therapy [7].

Patients whose ICP remained high with conventional treatment were included in the study. Before randomization of the patient to a study group, we required that patient to have received maximal medical treatment (first-level measures). In addition, we required a CT to have been conducted within 24 hours before inclusion of the patient in the study; intravenous administration of 0.7 g/kg mannitol 1 hour before randomization; or a plasmatic osmolarity measurement above 320 mOsm/kg, in order to ensure that hyperosmolar therapy had been optimized before inclusion.

### Randomisation

Randomization was based on a computer-generated list that intercollated the two drugs. Allocation was done by the intensive care unit physician who was on duty, once the patient had been found to meet the inclusion criteria and none of the exclusion criteria. Data collection and patient follow up were conducted by the same investigator (JPB).

### Blinding of treatment groups

The study was not blinded because it was difficult for us to mask treatment; thiopental is liophylized for administration and pentobarbital is not.

### Administration of barbiturates and monitoring of effects

Pentobarbital was administered in accordance with the protocol established by Eisenberg and coworkers [10], using a loading dose of 10 mg/kg over 30 minutes followed by a continuous perfusion of 5 mg/kg per hour for 3 hours. This was followed by a maintenance dosage of 1 mg/kg per hour.

Thiopental was administered in the form of a 2 mg/kg bolus administered over 20 seconds. If the ICP was not lowered to below 20 mmHg, then the protocol permitted a second bolus of 3 mg/kg, which could be readministered at 5 mg/kg if necessary to reduce persistently elevated ICP. The maintenance dosage was an infusion of thiopental at a rate of 3 mg/kg per hour.

In both treatment groups, for cases in which the maintenance dosage did not achieve the reduction in ICP to below the 20 mmHg threshold, the maintenance dosage for both drugs could be increased by 1 mg/kg per hour, while looking for electroencephalographic burst suppression or even the flat pattern, in order to ensure that different doses of the two barbiturates were equipotent. Electroencephalography was conducted daily in a noncontinuous manner (Nicolet; Viasys Healthcare, Verona Road, Madison, WI, USA). Results were analyzed by an experienced neurologist who was blinded to the treatment of the patients.

In those patients in whom barbiturate coma did not control ICP, we used decompressive craniotomy and/or external lumbar drainage, in accordance with the Munch criteria, as life-saving measures [11,12].

### Effectiveness criteria

Adequate response to treatment was defined as a decrease in ICP to below 20 mmHg, and maintenance below this threshold for at least 48 hours. To describe the ICP, we also followed the criteria previously employed by Stocchetti and coworkers [13]; the arithmetic mean of ICP data recorded during every 24-hour period, after filtering to exclude inaccurate readings, was calculated and expressed as 'mean ICP'. Three ICP blocks were considered for further analysis: less than 20 mmHg, 20 to 30 mmHg, and more than 30 mmHg.

Uncontrollable ICP was defined as follows: ICP of 21 to 35 mmHg for 4 hours, ICP of 36 to 40 mmHg for 1 hour, or ICP above 41 mmHg for 5 minutes, in the absence of external interventions. We also defined as unresponsive to treatment those cases in which, because of refractory ICP, the patient needed some other treatment (surgery and/or lumbar drainage) and cases in which the patient progressed to brain death.

Although it was not a main objective of the study, patients were evaluated 6 months after injury using the Glasgow Outcome Scale [14].

### Withdrawal of treatment

When ICP was controlled (<20 mmHg for 48 hours), we conducted a step-wise reduction in the barbiturate coma in steps that reduced the dosage by 50% every 24 hours until the infusion was suspended. In the event of ICP values rising to the study's inclusion values during the withdrawal of barbiturate treatment, the perfusion dosage was once again increased to achieve control of the patient's ICP.

### Sample size

Accepting an α error of 0.05 and a β error of 0.2 in a bilateral hypothesis contrast, we estimated that 47 patients were needed in each group to detect differences of 30% or greater in the control of ICH. To calculate sample size, we assumed that the therapeutic response rate in the pentobarbital group would be 50%, excluding patients lost to follow up.

### Statistical analysis

Quantitative variables are expressed as the mean and standard deviation from the mean (SD) in normal distributions, and as median and interquartile range in cases that were not normally distributed. Qualitative variables are expressed as percentages, along with 95% confidence interval (CI). To determine whether variables followed a normal distribution, we used the Shapiro Wilks test. For the comparison of quantitative variables, Student's *t*-test was used if the variable followed a normal distribution. In other cases, we used the Mann-Whitney U-test. For the comparison of qualitative variables, we used χ^2 ^or Fisher's exact test, as appropriate.

Given that the randomization did not create groups that were similar in terms of types of intracranial lesions shown on the CT results, which is a prognostic variable that influences the effectiveness of barbiturate treatment in controlling ICP, we conducted a multivariate analysis using binary logistic regression, so that we would include the prognostic variables with the most plausible association with the dependent variable 'uncontrollable ICP'. These are variables such as age, GCS score at admission, and the worst CT obtained within 24 hours before inclusion of the patient in the study, as well as the type of barbiturate administered. To achieve this multivariate analysis, and given the small number of cases in each of the five groups in Marshall's classification, the CT data were grouped into focal and diffuse lesions. We also included in the model the minimum daily MAP during barbiturate treatment, given that in the second and third days of treatment there were statistically significant differences between the groups in the univariate analysis. The significant variables identified by the 'likelihood ratio' ≤ 0.1 test were used, along with those whose inclusion affected the calculation of the effect of the 'treatment group' variable.

Both treatment groups were very similar in terms of other known prognostic variables, such as the presence of hypoxia, hypotension before hospital admission and pupil reactivity, and the univariate analysis did not identify differences between them, so these were not included in the multivariate analysis.

To analyze the variable ICP, which was determined on an hourly basis, we calculated the area under the curve (AUC) at 24, 48 and 72 hours, and also standardized by time [15].

For all comparisons, we considered statistical significance to have been achieved if the two-tailed α error probability was 5% or less (*P *≤ 0.05). Statistical analyses were conducted using SPSS version 15 (SPSS Inc., Chicago, IL, USA).

## Results

Preliminary results for the first 20 patients have already been published elsewhere [16].

From May 2002 to July 2007, 480 TBI patients were admitted to the intensive care unit of the Son Dureta University Hospital. Of these 480 patients, 71 (14.8%) presented with ICH refractory to first-level measures, of whom 44 were included in the study. The study was concluded prematurely because of the unexpected and slow inclusion rate; this could have modified some uncontrollable environmental factors that may affect results. The reasons for not including the remaining 27 refractory ICH cases were as follows: 13 patients were included in other studies, six were older than 76 years, five were admitted with nonreacting midriatic pupils and with clinical evidence of brain death, two presented with haemodynamic instability at the time of randomization, and one patient was transferred to a different hospital during the first 24 hours of admission, which excluded that patient from follow-up analysis. On average, the barbiturate coma was initiated in the thiopental group at 89 ± 15.5 hours after admission and in the pentobarbital group at 61 ± 14.3 hours after admission (*P *= 0.33).

The baseline characteristics of the 44 patients included in the study, 22 randomized to each group, are presented in Table 1. There were no statistically significant differences with respect to epidemiological data, co-morbidity (data not shown) or lesions associated with TBI, although there were differences in the CT classification.

**Table 1:** Baseline characteristics of patient population

Characteristic	Thiopental (n = 22)	Pentobarbital (n = 22)	*P*
Sex (male; n)	19	19	1
Age (years)	26 (20 to 41)	32 (22 to 43)	0.45
ISS	25 (24 to 34)	25 (25 to 38)	0.77
SAPS II	42 (28 to 54)	43 (38 to 46)	0.95
APACHE II	23 (15 to 25)	20 (18 to 26)	0.27
APACHE III	60 (38 to 73)	52 (32 to 76)	0.41
Associated lesion (n)			
Thoracic injury	7	2	0.13
Abdominal injury	4	1	0.34
Extremities injury	9	5	0.20
Admission CT (n)			
Diffuse injury without brain swelling	12	4	0.046
Diffuse bilateral brain swelling	6	12	
Diffuse unilateral brain swelling with midline shift	1	0	
Any mass lesion > 25 ml	3	6	

Age, ISS, SAPS II, APACHE II and APACHE III are expressed as median and interquartile range. APACHE, Acute Physiology and Chronic Health Evaluation; admission CT, admission computed tomography (according to the Traumatic Coma Data Bank); ISS, Injury Severity Score; SAPS, Simplified Acute Physiology.

The summary of prognostic variables for the 44 patients is shown in Table 2. As in Table 1 the characteristics of the worst CT conducted before inclusion in the study differed between the two groups.

**Table 2:** Prognostic variables of patient population

Variable	Thiopental (n = 22)	Pentobarbital (n = 22)	*P*
Admission GCS score	6.5 (3.0 to 7.2)	7 (4.7 to 10.0)	0.38
Out-of-hospital hypoxia (n)	5	7	0.63
Out-of-hospital hypotension (n)	5	4	1
Pupillary reactivity (n)^a^			
One reacting	3	5	0.66
Both reacting^b^	12	14	
Pre-enrolment CT (n)			
Diffuse injury without brain swelling	8	5	0.04
Diffuse bilateral brain swelling	1	8	
Diffuse injury unilateral brain swelling with midline shift	5	1	
Any mass lesion evacuated	7	5	
Nonevacuated mass lesion	1	3	

Admission GCS is expressed as median (interquartile range). ^a^Pupillary reactivity at hospital admission. ^b^Miotic pupils were considered as reactive. CT, computed tomography; GCS, Glasgow Coma Scale.

### Effectiveness criterion: control of intracranial pressure

The distribution of the ICP during the first 3 days of treatment, according to Stocchetti's criteria, is summarized in Table 3. The missing cases during these 3 days were due to brain deaths or to receipt of rescue treatment for uncontrollable ICP. Finally ICP was uncontrollable in 11 cases (50%) in the thiopental group and in 18 patients (82%) in the pentobarbital group (*P *= 0.03). In nonresponding patients, we chose to place a lumbar drainage in five, in three we opted for surgical treatment, and in three other patients we combined both treatments, drainage and surgery. Surgical decompression was conducted in four patients in the thiopental group and in two of the patients in the pentobarbital group. The number of hyperosmolar treatments administered (manitol and/or hypertonic saline) during the barbiturate coma was similar in both groups: 16.5 (8.0 to 24.2) in the thiopental group and 16.5 (3.0 to 21.5) in the pentobarbital group (*P *= 0.9). The mean ± SD duration of the barbiturate coma was 156 ± 60 hours for thiopental and 108 ± 100 hours for pentobarbital (*P *= 0.06). Seven (31.8%) patients presented an ICP rebound with thiopental and six (27.3%) with pentobarbital (*P *= 0.74) during treatment withdrawal.

**Table 3:** Mean ICP recorded per day during the first 3 days of barbiturate coma

Drug	Day	Mean ICP (n[%])					
		
		<20 mmHg		20 to 30 mmHg		>30 mmHg	
Thiopental	1	10	46	11	50	1	5
	2	15	71	4	19	2	10
	3	12	63	6	32	1	5
Pentobarbital	1	9	41	8	36	5	23
	2	8	38	9	43	4	19
	3	6	43	8	57	0	0

Mean intracranial pressure (ICP) is the arithmetic mean of ICP data recorded during every 24-hour period, according to Stocchetti's criteria [13]. Data are presented as number of cases and as a percentage of the total number of cases each day.

**Figure 1:**
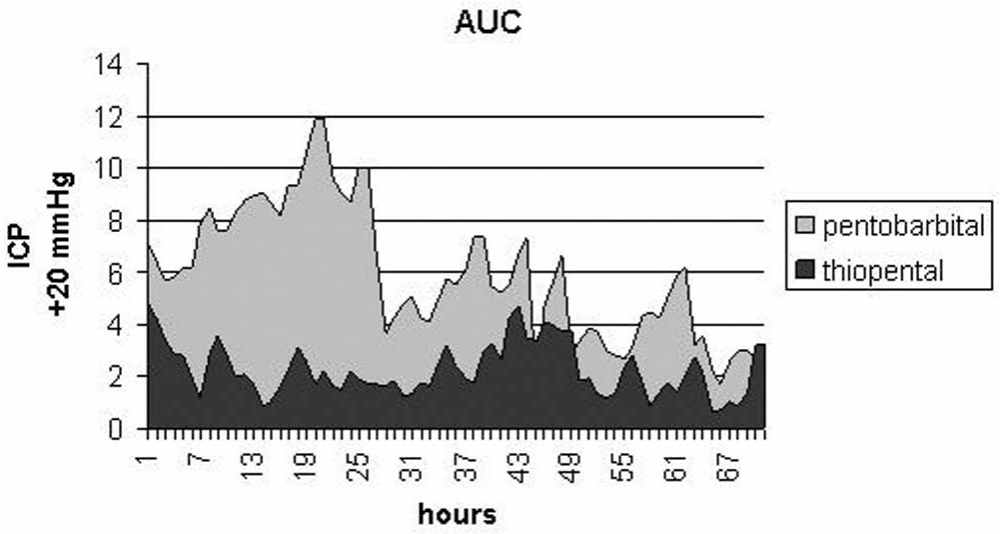
AUC of ICP data. Presented are areas under the curve (AUCs) of the intracranial pressure (ICP) data, standardized by time, with a base value of 20 mmHg.

Figure 1 presents the AUC for ICP above 20 mmHg, standardized over time, as follows. The ICP value of AUC_0–24 h _was 458.00 mmHg·hour (95% CI = 421.84 to 494.16) in the thiopental group and 550.63 mmHg·hour (95% CI = 411.31 to 689.95) in the pentobarbital group. The AUC_0–48 h _was 913.18 mmHg·hour (95% CI = 814.08 to 1,012.27) in the thiopental group and 997.27 mmHg·hour (95% CI = 757.10 to 1,237.43) in the pentobarbital group. The AUC_0–72 h _in the thiopental group was 1,291.69 mmHg·hour (95% CI = 1,172.27 to 1,411.12) and 1,399.73 mmHg·hour (95% CI = 1,291.11 to 1,508.35) in the pentobarbital group. Standardized over time, the AUC per hour in the thiopental group was 23.90 mmHg (95% CI = 22.00 to 25.81) and in the pentobarbital group it was 29.39 mmHg (95% CI = 23.20 to 35.59).

Both treatment groups were similar in terms of known prognostic variables, such as presence of hypoxia, hypotension before hospital admission and pupil reactivity, and the univariate analysis did not identify differences between them. Therefore, these were not included in the multivariate analysis. The logistic regression analysis showed that, after adjusting for the worst CT and the type of barbiturate used, thiopental was five times more likely than pentobarbital to control ICP (odds ratio = 5.1, 95% CI = 1.2 to 21.9; *P *= 0.027). The Hosmer-Lemeshow test indicated that the fit of the model was good (*P *= 0.799). The association of focal lesions in the pre-inclusion CT with ICP control was 3.6 times higher than that for the diffuse lesions. The relative risk for good control of ICP in the thiopental versus pentobarbital group was 2.26 for patients with focal lesions and 3.52 for those who presented with diffuse lesions. The other variables analyzed did not exhibit a significant relationship to ICP control, and did not modify the effect of the barbiturate treatment, including the third day minimum MAP, which was significantly different between the two treatments (data not shown).

### Adverse side effects during the barbiturate coma

The secondary effects during the barbiturate coma are presented in Table 4. In both groups almost all patients presented with at least one MAP measurement below 80 mmHg. There were no differences between groups with respect to the incidence of infections, Sepsis related Organ-Failure Assessment (SOFA) scores before initiation of treatment, or the maximum SOFA value [17] during the entire period of barbiturate coma.

**Table 4:** Adverse events during barbiturate coma

Adverse event	Thiopental (n = 22)	Pentobarbital (n = 22)	*P*
Hypotension^a^	21	20	1
Respiratory infection^b^	18	17	1
Urinary infection^c^	0	2	0.49
Positive blood culture	4	1	0.34
ICP catheter colonization	7	5	0.5
CNS infection (CSF)^d^	3	0	0.23
SOFA pre^e^	7 (4.5 to 9.5)	8.0 (5.5 to 9.0)	0.57
SOFA maximum^f^	11 (10 to 12)	11 (10 to 12)	0.94

^a^Hypotension is defined as detection of a medium arterial blood pressure below 80 mmHg at any time during barbiturate coma. ^b^Respiratory infection: presence of a positive sputum culture. ^c^Urinary infection: presence of a positive urine culture. ^d^Central nervous system infection (CNS) infection (cerebrospinal fluid [CSF]): infection of the CNS with a positive culture in the CSF. ^e^SOFA pre: value of the Sepsis related Organ-Failure Assessment (SOFA) score before the beginning of the barbiturate coma. ^f^SOFA maximum: maximum value of the SOFA during the barbiturate coma, according the indication by Moreno and coworkers [16].

A thermodilution catheter was placed in 42 patients to facilitate haemodynamic control. The haemodynamic changes produced during the barbiturate coma are presented in Table 5. Differences of note include the minimum MAP, the pulmonary wedge pressure value, and the maximum norepinephrine dosage on days 2 and 3.

**Table 5:** Systemic changes during barbiturate coma

Parameter	Pretreatment	1st day	2nd day	3rd day	4rd day
Cardiac output (l/minute)^a^					
Thiopental	6.8 ± 1.4	6.4 ± 1.5	6.0 ± 1.4	6.7 ± 1.5	6.6 ± 1.8
Pentobarbital	7.4 ± 2.2	7.1 ± 1.9	6.5 ± 2.0	7 ± 1.4	6.1 ± 1.3
Cardiac index (l/minute per m^2^)					
Thiopental	3.6 ± 0.6	3.4 ± 0.6	3.1 ± 0.6	3.6 ± 1.6	3.5 ± 0.9
Pentobarbital	3.8 ± 1.2	3.8 ± 0.8	3.4 ± 0.8	3.6 ± 0.7	3.2 ± 0.6
Peripheral venous resistance (dines/m^2^)					
Thiopental	1,015 ± 325	1,022 ± 347	1,140 ± 429	1,089 ± 289	1,029 ± 253
Pentobarbital	952 ± 257	893 ± 210	1,003 ± 322	939 ± 261	914 ± 188
Pulmonary artery wedge pressure (mmHg)					
Thiopental	10.4 ± 4.5	9.6 ± 3.6	10.1 ± 4.1*	10.9 ± 4.6*	11.4 ± 3.5*
Pentobarbital	11.6 ± 4.0	11.4 ± 3.1	12.8 ± 3.1	13.2 ± 2.1	13.9 ± 3.1
mBP (mmHg)^b^					
Thiopental	92 ± 11	75 ± 7	76 ± 9*	76 ± 6*	76 ± 8
Pentobarbital	94 ± 10	74 ± 1	68 ± 10	70 ± 1	70 ± 10
NAD (μg/kg per minute)^c^					
Thiopental	0.18 ± 0.33	0.28 ± 0.27	0.37 ± 0.3*	0.46 ± 0.39	0.56 ± 0.63
Pentobarbital	0.19 ± 0.18	0.55 ± 0.68	0.73 ± 0.69	0.60 ± 0.44	0.96 ± 0.79
PO_2_/FiO_2_^d^					
Thiopental	284 ± 130	300 ± 139	293 ± 132	285 ± 138	254 ± 119
Pentobarbital	317 ± 127	304 ± 116	262 ± 125	211 ± 77	184 ± 92
Haemoglobin					
Thiopental	10.9 ± 1.6	10.7 ± 1.3	11 ± 1.2	10.8 ± 0.9	10.7 ± 1.4
Pentobarbital	10.6 ± 1.2	10.1 ± 1.0	10.4 ± 1.1	10.5 ± 1.1	10.2 ± 1.2
Temperature (°C)^e^					
Thiopental	35.8 ± 0.5	34.6 ± 1.3	34.6 ± 3.4	34.9 ± 1.0	34.9 ± 1.0
Pentobarbital	35.7 ± 1.0	34.6 ± 1.2	34.3 ± 1.3	34.4 ± 1.3	34.2 ± 1.1

^a^Cardiac output, cardiac index, peripheral venous resistance and pulmonary artery wedge pressure: the values are the mean values over 24 hours. ^b^mBP: minimum value of the medium blood pressure during the day. ^c^NAD: maximum dose of Noradrenaline bitartrate during the day. ^d^PO_2_/FisO_2_: ratio of partial oxygen tension to inspired fractional oxygen tension at 8:00 am. ^e^Temperature: value of the minimum central temperature. *P < 0.05.

### Six-month outcomes

In the thiopental group, the neurological outcomes at 6 months (in accordance with Glasgow Outcome Scale score) were as follows: death in nine patients, vegetative state in two, severe disability in two, moderate disability in four and good recovery in four. In the pentobarbital group, the 6-month outcome was death in 16 patients, vegetative state in one, moderate disability in two and good recovery in two. In both groups one case was missing from the 6-month follow up analysis

## Discussion

The results of this study indicate that thiopental is five times more effective than pentobarbital in controlling refractory ICH. However, these findings must be interpreted with caution, given the small sample size and the fact that the study was unable to mask assignment to treatment groups.

Barbiturate coma is at present the only therapy for which we have class II evidence, under BTF Guidelines [1], of efficacy in treating refractory ICH. Hence, it is perhaps the case that barbiturate coma is the most used second-level measure, with a usage frequency reported in the literature that varies from 13% to 56% [18,19]. Therefore, it is important to test the effectiveness of the various barbiturates available for controlling ICP refractory to first-level measures.

### Differences between oxibarbiturates and thiobarbiturates

The pharmacokinetic characteristics of thiopental and pentobarbital are different because their protein binding, distribution volume and clearance differ [20]. Nevertheless, the mean half life (thiopental 6 to 46 hours and pentobarbital 15 and 48 hours), which is the fundamental pharmacological parameter, differs little between the two agents. It therefore does not appear that these pharmacokinetic differences have clinical repercussions.

One difference between these two groups of barbiturates is the presence of active metabolites. Thiopental has five metabolites, of which four are inactive and one (pentobarbital, or pentobarbitone) is active. Therefore, pentobarbital is an active metabolite of thiopental. This fact, along with the great intra-individual and inter-individual variability in the metabolism of these agents (caused by the existence of enzymatic induction phenomena associated with hepatic cytochrome P450), results in a weak correlation between serum concentrations and pharmacological effect. For this reason, monitoring this treatment with electroencephalography is strongly recommended.

At the experimental level, various studies have compared these two medications. Hatano and coworkers [21], in a study conducted in a dog model, concluded that thiobarbiturates provoke cerebral vasoconstriction, which could help to redistribute cerebral blood flow toward ischaemic zones. Cole and colleagues [4] demonstrated that thiopental reduced the size of the ischaemic area more than did pentobarbital, even though both drugs achieved electroencephalographic burst suppression patterns. Shibuta [5] observed that thiopental, but not pentobarbital, was capable of limiting the cytotoxic damage caused by nitric oxide. Almaas and coworkers [3] observed that the different barbiturates had different neuroprotective effects with respect to oxygen and glucose deprivation in a model using human neurone cultures. Thiopental exhibited a neuroprotective effect at all the dosages studied, whereas pentobarbital was neuroprotective only at elevated dosages. Finally, in an *in vitro *study, Smith and colleagues [6] demonstrated that although thiopental provoked 96% inhibition of lipid peroxidation, pentobarbital had almost no effect.

These experimental studies demonstrate that not all barbiturates are equal and that their neuroprotective capacity and effectiveness may differ [22]. Therefore, despite the unavoidable methodological limitations of the present study, we believe that our results may have clinical relevance.

### Secondary effects of barbiturate coma

The most frequently detected secondary effect in our study, as might be expected, was arterial hypotension, which occurred in 21 patients in the thiopental group and 20 patients in the pentobarbital group. Although this incidence may be greater than that in previous studies [10], we attribute this to the definition of hypotension used (detection at any time in the barbiturate coma of MAP < 80 mmHg), which did not take the 'time' variable into account. For that reason, we collected data on other variables, such as maximum daily norepinephrine dosage and minimum daily MAP. Nearly all patients were monitored using a pulmonary artery thermodilution catheter, and arterial hypotension episodes were rigorously managed with fluid therapy and vasoactive drugs.

We would note that the changes produced by pentobarbital at the cardiac and respiratory level were, in general terms, greater than those produced by thiopental. This is because (as shown in Table 5) cardiac output, cardiac index, and partial oxygen tension/fraction of inspired oxygen ratio exhibited greater changes during treatment with pentobarbital than with thiopental. This observation contrasts with the findings of previous experimental studies [23], in which it appeared that at high doses pentobarbital was safer and better tolerated than thiopental.

Other complications (mostly infections) and the incidence of multiple organ dysfunction (identified using maximum SOFA) were similar in the two groups.

### Limitations of the study

As previously noted, this study has two important limitations. First, it was not a blinded study because the pentobarbital was not liophylized and thiopental was. Second, the sample size was small, so that small changes in the principal variable studied, namely ICP control, could significantly affect the statistical analysis.

The classical view is that ICP response to barbiturates varies from 30% to 50%, and so it is possible that part of the difference found between drugs is due to poor response by the pentobarbital group as a result of any confounding bias. The randomization process is a potent mechanism that tends to eliminate bias by randomly distributing the values of all of the variables to the experimental groups. Nonetheless, the tool is not perfect and the groups frequently exhibit an imbalance in some confounding variable, especially when working with samples that are not very large. For this reason, in this study we used logistic regression analysis to eliminate any possible bias, and separate, independent analyses of the CT data were also conducted by two investigators who did not know the experimental group to which the patients belonged.

Another limitation is that the dosages in the two groups were not the same. This leaves the possibility that the reason for the difference between agents that we identified is inadequate pentobarbital dose. Although in the two groups barbiturates were used with the end-point of ICP control, in this type of patient we also employ daily noncontinuous electroencephalographic monitoring. In this way, we believe that – despite different doses – the effect of the two barbiturates can be considered as equipotent because we looked for burst suppression or even the flat electroencephalographic pattern if the ICP was not controlled and the patients remained haemodynamically stable.

## Conclusion

In this patient sample, thiopental appeared to be more effective than pentobarbital in controlling ICH refractory to first-level measures, according to the BTF Guidelines. Nevertheless, these findings should be interpreted with caution because of the imbalance in CT characteristics and the different dosages employed in the two arms of the study. However, the present study is useful as a hypothesis testing exercise and will help to inform the design of future studies. These findings corroborate experimental evidence suggesting that there are differences in the neuroprotective mechanism between the two treatments, and this study may be a first step toward translating evidence from animal models to clinical disease.

The incidence of secondary effects during treatment was similar between groups.

## Key messages

• High doses of barbiturates are used in those patients with severe TBI who present with refractory ICH, and this recommendation is included in the BTF Guidelines.

• Until now no controlled studies have been conducted to compare the effectiveness of pentobarbital and thiopental in controlling refractory ICH. Nevertheless, at the experimental level, research has demonstrated that their mechanisms and levels of neuroprotection differ.

• Thiopental appeared to be more effective than pentobarbital in controlling ICH refractory to first-tier measures, although these results should be interpreted with caution because of the imbalance in CT characteristics and other limitations of the study.

## Abbreviations

AUC: area under the curve; BTF: Brain Trauma Foundation; CI: confidence interval; ICH: intracranial hypertension; ICP: intracranial pressure; MAP: mean arterial pressure; SD: standard deviation; SOFA: Sepsis related Organ-Failure Assessment; TBI: traumatic brain injury.

## Competing interests

The authors declare that they have no competing interests.

## Authors' contributions

JPB was responsible for study design, acquisition of data, analysis and interpretation of data, and writing of the manuscript. JALP was responsible for acquisition of data and patient randomization. JH acquired data and conducted patient randomization. JMA acquired data and conducted patient randomization. JMR conducted statistical analyses. GF conducted statistical analyses. MB was responsible for designing the study and reviewing CT findings. JI was responsible for designing the study and reviewing CT findings. JI revised the article critically and gave final approval to the version to be published.
